# Progress in State Medicine

**Published:** 1903-10-31

**Authors:** 


### Progress in State Medicine.

(*Concluded from page* 69.)

TUBERCULOSIS—*continued.*

Bulstrode[6] says that, whilst it is impossible to accurately state the number of sanatoria which at present exist in England, by excluding Poor Law institutions, and including hospitals for consumptives, and homes for the dying, there would appear to be, approximately speaking, about 60 institutions, public or private, at which cases of pulmonary tuberculosis may be accommodated. The number of beds which are embraced by these institutions is probably under 2,500, and assuming each bed to be occupied for three months, there may be in England and Wales, Ireland and Scotland, some 10,000 beds available annually. If to this number the accommodation available at workhouses and infirmaries be added, the total must be very materially augmented. The main object in the provision of sanatoria is to secure means whereby the tuberculosis patient may be taken out of the environment in which he either contracted or developed his illness, and to put him under conditions in which he may cease to furnish hospitality to the tubercle bacillus. Such conditions can be provided almost as well in a small suitably-constructed establishment as in a large three-storied institution, the adequate ventilation of which is somewhat difficult of attainment, and hence he questions the desirability of waiting until a marble palace can be provided, and in the meantime allowing an uncertain number of people to die whose lives might otherwise be prolonged. He quotes the words of Petit: "In all sanatoria, whatever their geographical position, the curative results obtained are nearly the same, provided that the hygienic method is applied there in all its scientific rigour." It is far more important to get control of cases which are likely, owing to their domestic environment, to be some danger to the community, than cases which are in the earlier stages of the disease, and who are expectorating little, if at all. He states that Rushton Parker thinks that about 35 out of the 50 or 60 deaths which occur annually in Westmorland from tuberculosis might, were there an institution for advanced cases, die therein. He also thinks that a home with 20 beds would suffice for 40 cases remaining six months each, and he calculates the total cost per annum to be about £1,300, or £65 a bed. About one-sixth of the cases would, he thinks, be chargeable to the guardians. The County Council has legal power to provide such a home, and the cost thereof would be covered by a penny rate. He points out that the 8,000 consumptives in the English workhouse infirmaries cost £600,000 a year, or £75 per head. Bulstrode mentions three chief agencies upon which he would rely as regards the control of pulmonary tuberculosis, assuming always a sufficiency of food. (1) The education of older children in the principles of health and the periodical physical examination of all school children with a view to improving the health of those prone to tuberculosis, or who may already be suffering from the disease in an unrecognised though not unrecognisable form. (2) Some such system of compulsory insurance against sickness and invalidity as obtains in Germany. (3) Better housing and improved conditions of employment of the working classes—*i.e.*, more light, more air-space, better ventilation, and greater cleanliness in the home, workshop, and factory. Kirstein[7] carried out a series of experiments to determine the length of time during which pathogenic bacteria which are associated with particles of dust or minute drops of moisture may remain alive in rooms subjected to ordinary illumination with diffuse sunlight. Experiments with the tubercle bacillus showed that this organism preserved its vitality when thrown out in moisture in the form of the finest possible particles for at least four days. Tubercle bacilli which had been thrown out in a spray in that manner and collected eight days after the spraying were found to have lost their virulence. The period at which the tubercle bacilli die under conditions mentioned is, therefore, between four and eight days. The experiments of other authors along this line with the tubercle bacillus are discussed. It was argued that the great variation in results obtained is due largely to the fact that the tubercle bacilli were encased in drops of moisture or sputum of very different size in different experiments. Kirstein found that where tubercle bacilli were protected from the action of diffuse sunlight and desiccation, in particles of sputum of considerable size, the organisms preserved their vitality for a period of three months or more.

[6] Lancet, Aug. 15, 1903. [7] Public Health, September, 1903.

